# Temporal and Effort cost Decision-making in Healthy Individuals with Subclinical Psychotic Symptoms

**DOI:** 10.1038/s41598-018-38284-x

**Published:** 2019-02-15

**Authors:** Damiano Terenzi, Elena Mainetto, Mariapaola Barbato, Raffaella Ida Rumiati, Marilena Aiello

**Affiliations:** 1 Area of Neuroscience, SISSA, Trieste, 34136 Italy; 2 Zayed University, Dubai, 19282 United Arab Emirates; 3 ANVUR, Roma, 00153 Italy

## Abstract

The value people attribute to rewards is influenced both by the time and the effort required to obtain them. Impairments in these computations are described in patients with schizophrenia and appear associated with negative symptom severity. This study investigated whether deficits in temporal and effort cost computations can be observed in individuals with subclinical psychotic symptoms (PS) to determine if this dysfunction is already present in a potentially pre-psychotic period. Sixty participants, divided into three groups based on the severity of PS (high, medium and low), performed two temporal discounting tasks with food and money and a concurrent schedule task, in which the effort to obtain food increased over time. We observed that in high PS participants the discounting rate appeared linear and flatter than that exhibited by participants with medium and low PS, especially with food. In the concurrent task, compared to those with low PS, participants with high PS exerted tendentially less effort to obtain snacks only when the required effort was high. Participants exerting less effort in the higher effort condition were those with higher negative symptoms. These results suggest that aberrant temporal and effort cost computations might be present in individuals with subclinical PS and therefore could represent a vulnerability marker for psychosis.

## Introduction

In everyday decision-making, we are frequently asked to make decisions concerning the value of rewards. These decisions require us to take into account both the temporal cost (how long we need to wait) and the effort cost (how much effort we need to expend) to obtain a reward^1–4^. Research has shown that humans devalue rewards associated with delays similarly to the way in which they devalue those associated with effort^5,6^, but that distinct brain networks respond to delayed reward and effort costs^7,8^. Specifically, the increasing subjective value of a delayed reward is associated with an increased activity of the ventral striatum and the ventromedial prefrontal cortex, whereas the increasing subjective value of an effortful reward is associated with a decreased activity of the anterior cingulate cortex and the anterior insula^7^.

Abnormal temporal and effort discounting of rewards characterizes several forms of psychopathology, including schizophrenia^3,9^. By and large, individuals with schizophrenia seem to choose immediate over long-term rewards more frequently than healthy controls, whether in monetary choice questionnaires^10,11^ or intertemporal choice tasks^12,13^. However, although impaired working memory has frequently been found associated with higher discounting rates^10,11^, cognitive dysfunction alone does not seem to fully account for the abnormal discounting of rewards observed in these patients. It has been reported that patients also exhibit less willingness to work to obtain monetary rewards when a high level of effort is required, a result that has been observed across different experimental paradigms^14,15^. The devaluation of rewards associated with delays or effort has been found to correlate with the severity of negative symptoms, in particular avolition and anhedonia^11,16^ (but see ref.^17^ for a different result), while there is no evidence of a link with positive symptoms such as hallucinations and delusions^11,16^. Over the years, studies have shown that the increasing severity of negative symptoms corresponds to steeper temporal and effort discounting of rewards in these patients. Overall, these alterations have been associated with functional abnormalities in brain reward-related regions such as orbitofrontal and/or anterior cingulate cortex^16,18^, and have been interpreted as a result of a deficit in the representation of value of rewards^19^, especially concerning aspects such as reward prediction, anticipation and valuation. This evidence accords well with the Incentive Salience Hypothesis, proposed by Berridge, whereby the ability to experience pleasure during reward consumption (liking), and the motivation to obtain a reward (wanting or incentive salience) are two different constructs sub-served by distinct neural pathways^20^. In particular, dopamine is critical for wanting, but not liking, and plays a role in signaling incentive salience and initiating appetitive behavior^20,21^. In line with this theory, schizophrenia does not seem to be associated with diminished capacity to experience pleasure^22^.

Aberrant motivation and discounting of rewards has also been observed within the psychosis continuum. For instance, steeper temporal and effort discounting of rewards has been found, although not consistenly^23^, in individuals with schizotypal personality traits^24–26^. Also in individuals who were putatively prodromal for psychosis, studies have found a reduced motivation to obtain rewards and an altered activity in reward related regions when they perform reward tasks^27,28^. Taken together these results suggest that aberrant temporal and effort discounting of rewards might be a vulnerability marker for psychosis. To corroborate this hypothesis, the need to further explore these mechanisms with other “at-risk” populations along the continuum is warranted. The term psychotic-like experiences refers to subclinical psychotic symptoms experienced by healthy individuals, who, in the absence of a clinical threshold of psychosis, may share a degree of overlap in clinical manifestations with individuals with clinical psychosis^29–31^. Interestingly, a study of 37 healthy participants with varying levels of subclinical psychotic symptoms observed that dysfunctional activity in the ventral striatum during a task assessing anticipation of monetary gains and losses was dependent on the degree of symptom expression^32^. However, this study did not included direct measurement of temporal and effort discounting of rewards in this population.

In the present study we investigated whether alterations of both temporal and effort computations are present in individuals with subclinical psychotic symptoms, by using a temporal discounting task and a concurrent schedule task, which assesses the willingness to exert effort to obtain a reward. Stimuli of the concurrent task were selected according to a liking rating task that also allowed us to obtain liking scores. In the first task, we used both monetary and food rewards, while in the second task we used only food rewards. We opted for this type of reward because, especially in the concurrent task, a primary reward such as food is best suited to disentangle the liking and wanting processes^7^. We used the Community Assessment of Psychic Experiences (CAPE^33^) to identify individuals with subclinical psychotic symptoms. This self-report measure assesses the frequency and distress of psychotic-like feelings, thoughts, or mental experiences related to three domains: positive, negative and depressive symptoms. From a large sample of 334 healthy participants who were screened for psychotic-like experiences, we selected three groups based on the level of psychotic symptoms (high PS, medium PS and low PS) using cut-offs on positive symptoms provided by the CAPE^33^.

Based on the literature on schizophrenia patients, we hypothesized that individuals with high and/or with medium PS might choose immediate over long-term rewards more frequently compared to individuals with low PS, and to be less willing to exert effort in the concurrent schedule task. Furthermore, we expected that a steeper temporal discounting of rewards and/or a decreased willingness to expend effort to obtain rewards would be associated with negative but not positive symptoms severity.

## Results

### Demographic and clinical data

Individuals with high PS, medium PS and low PS were matched for gender, age, education and BMI (*ps* > 0.07). In addition, they did not differ significantly on subjective ratings of hunger (*ps* > 0.34). Participants with high PS and medium PS scored significantly higher than participants with low PS on all the CAPE sub-scales (all *ps* < 0.01). Compared to those with medium PS, participants with high PS scored higher on the CAPE positive and negative symptoms sub-scales (respectively, *p* < 0.001 and *p* < 0.05) but not in the depression sub-scales (all *ps* > 0.07). No differences between groups emerged when the BDI-II scale, the BIS-11, the TEPS and the BAS questionnaires were analyzed (all *ps* > 0.1). Furthermore, no differences emerged in the Digit Span forward test (*p* = 0.76) and in the Stroop Test for both performance time and number of errors (all *ps* > 0.73). For demographical and clinical information see Table 1. See supplementary information for detailed statistics.

**Table 1:** Demographic, neuropsychological and questionnaire data (mean and standard deviation).

	high PS (n = 19)	medium PS (n = 21)	low PS (n = 20)
Gender (female)	14	16	9
Age (y)	23.47 (2.91)	23.09 (3.3)	23.75 (2.71)
Education (y)	14.05 (2.27)	14.19 (1.8)	14.55 (1.85)
BMI	23.14 (2.56)	21.82 (3.45)	21.89 (2.6)
**Psychosis Proneness (CAPE):**			
Positive symptoms	3.73 (0.53)* ^≠^	2.96 (0.12)^≠^	2.42 (0.2)
Positive frequency	1.88 (0.23)* ^≠^	1.57 (0.1)^≠^	1.3 (0.15)
Positive distress	1.85 (0.36)* ^≠^	1.39 (0.12)^≠^	1.12 (0.11)
Negative symptoms	4.82 (0.91)* ^≠^	4.25 (0.79)^≠^	3.4 (0.86)
Negative frequency	2.27 (0.44)* ^≠^	2.01 (0.35)^≠^	1.68 (0.37)
Negative distress	2.55 (0.52)* ^≠^	2.23 (0.48)^≠^	1.72 (0.51)
Depressive symptoms	5.22 (0.95)^≠^	4.69 (0.9)^≠^	3.77 (0.86)
Depressive frequency	2.37 (0.46)^≠^	2.14 (0.36)^≠^	1.83 (0.89)
Depressive distress	2.85 (0.56)^≠^	2.55 (0.6)^≠^	1.94 (0.54)
**Cognition:**			
Digit Span Forward	6.74 (1.1)	6.71 (1.06)	6.95 (1.15)
Stroop: time (s)	9.7 (5.56)	10.27 (5.07)	9.27 (5.81)
Stroop: errors	0.14 (0.42)	0.17 (0.45)	0.07 (0.24)
**Mood:**			
BDI-II	12.16 (6.98)	10.19 (7.33)	7.35 (5.7)
**Reward sensitivity – impulsivity:**			
BAS total	41.48 (4)	41.19 (4.74)	41.2 (3.68)
BAS reward responsiveness	18.84 (1.26)	18.48 (1.75)	17.75 (1.68)
BAS drive	11.74 (1.88)	12.09 (2.12)	12 (2.15)
BAS fun-seeking	10.89 (2.82)	10.62 (2.4)	11.45 (1.85)
TEPS anticipatory	44.58 (4.07)	45.52 (4.61)	44.15 (5.57)
TEPS consummatory	36.47 (4.73)	37.81 (4.27)	35.4 (4.08)
BIS total	65.26 (9.46)	61.57 (9)	60 (7.75)
BIS attentional	17.58 (3.42)	17.09 (2.84)	15.8 (2.63)
BIS motor	22.42 (4)	19.9 (3.67)	20.3 (3.93)
BIS non-planning	25.26 (4.01)	24.57 (4.54)	23.9 (3.04)

*Significantly different from medium PS, *p* < 0.05; ≠significantly different from low PS, *p* < 0.05;

y = years; s = seconds; BMI = body mass index; BDI-II = Beck depression Inventory; BAS = Behavioral Activation Scale; BIS = Behavioral Inhibition Scale; TEPS = Temporal Experience of Pleasure Scale; CAPE = Community Assessment of Psychic Experience.

### Temporal discounting tasks

The ANOVA on AUC with Group (high PS, medium PS, low PS) × Task (food, money) yielded a significant main effect of Task (F_1, 57_ = 8.06, *p* < 0.01), meaning a steeper temporal discounting for food than money. No other significant results emerged (*ps* > 0.68). The comparison among linear, hyperbolic and exponential discounting models showed that the linear model fitted the data better than the other models across rewards and groups (see Table 2).

**Table 2:** R^2^ values for Linear, Hyperbolic and Exponential model fits across rewards and groups.

	Money			Food		
	Linear	Hyperbolic	Exponential	Linear	Hyperbolic	Exponential
High PS	0.9	0.7	0.5	0.9	0.5	0.3
Medium PS	0.8	0.9	0.6	0.9	0.8	0.5
Low PS	0.9	0.8	0.6	0.9	0.7	0.3
Overall	0.9	0.8	0.6	0.9	0.6	0.4

**Figure 1:**
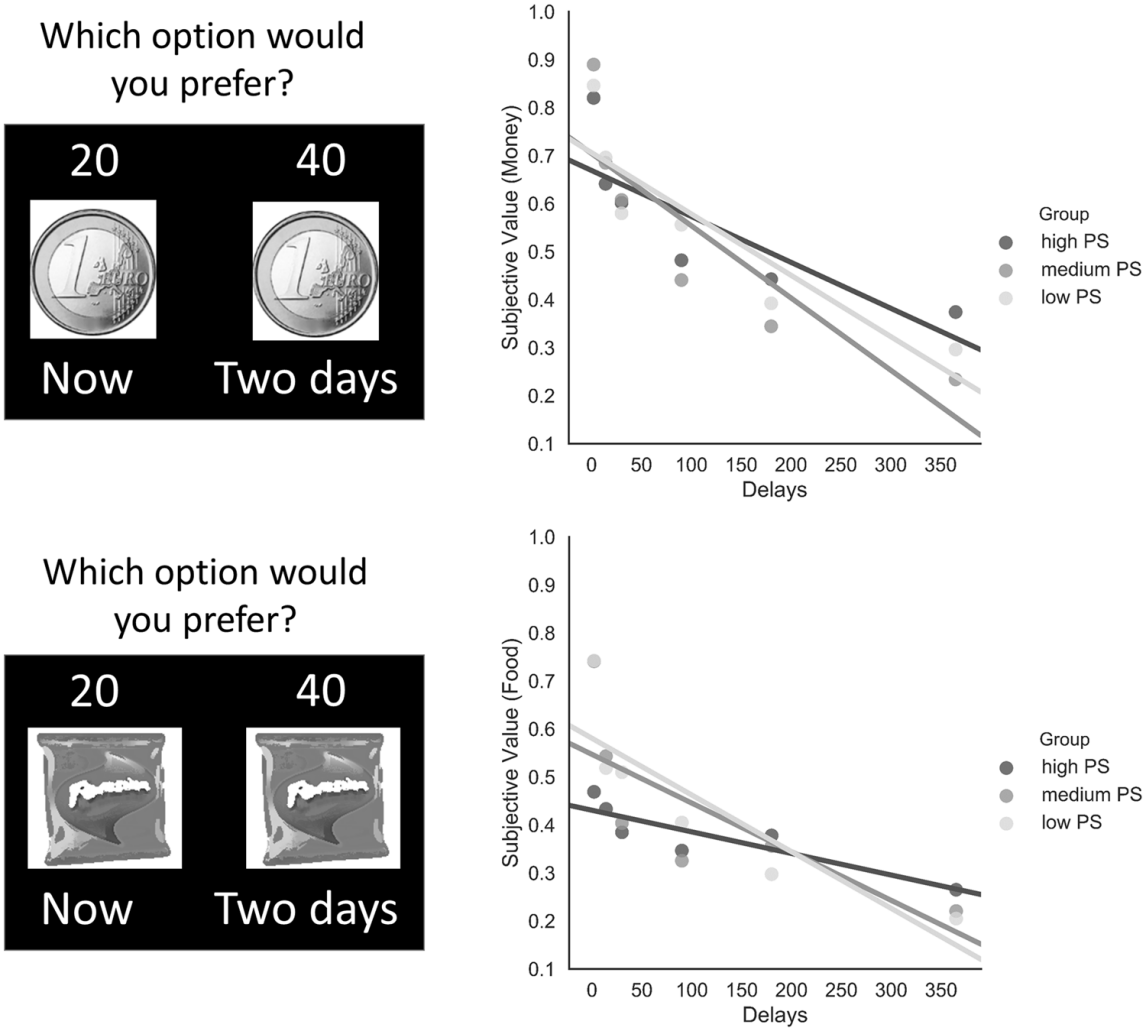
Temporal discounting tasks (left panel). Mean subjective values for money and food for high PS, medium PS and low PS (right panel). Lines represent the slope of the linear regression between subjective value and time intervals (delays).

The ANOVA on the slope values of the linear regression yielded a significant main effect of Task (F_1, 57_ = 10.61, *p* < 0.01) and a main effect of Group (F_2, 57_ = 8.21, *p* < 0.001). Post-hoc analysis showed a less steep slope for individuals with high PS compared to individuals with medium PS (*p* < 0.01) and low PS (*p* < 0.01) (see Fig. 1). There was no Group x Task interaction (F_2, 57_ = 1.44, *p* = 0.24). See supplementary information for statistics on hyperbolic parameter k.

### Concurrent schedule task

**Figure 2:**
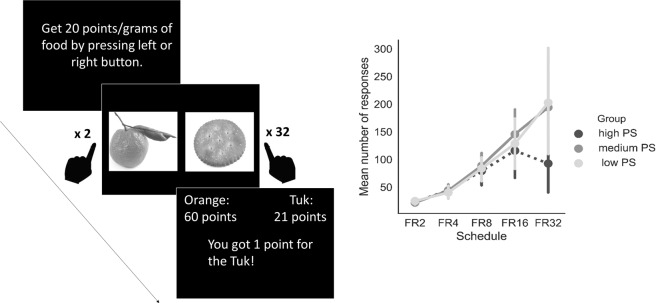
Concurrent schedule task. Example trial sequence of the FR32 schedule (left panel). Mean number of responses for snacks per schedule for high PS, medium PS and low PS subjects (right panel). FR refers to fixed ratio, which is the number of responses required to earn a single-snack point. FR for low-calorie items stay at 2 (FR2), whereas the FR for snack increases during the task (from FR2 to FR32). The error bars represent SEM. Representative images of food used in the experiment. Images have been taken from^62^.

The ANOVA on liking rating scores yielded a significant main effect of type of Food (F_1, 57_ = 24.21, *p* < 0.001), meaning a higher liking for high-calorie items than low-calorie items. No other significant results emerged (*Ps* > 0.6). During the concurrent schedule task, participants did not differ on number of key presses for the snack option in the following FR2, FR4, FR8 and FR16 schedules (all *ps* > 0.4). However, in the FR32 schedule the number of key presses was lower for the high PS group compared to the low PS group close to being statistically significant (*U* = 114, Z = −1.94, *p* = 0.052) (see Fig. 2). There were no differences in this schedule between individuals with high PS and medium PS (*U* = 132.5, Z = −1.81, *p* = 0.07) and between individuals with medium PS and low PS (*U* = 196, Z = 0.09, *p* = 0.92). Furthermore, participants did not differ on slope values (all *p* > 0.06). See supplementary materials for detailed statistics.

### Correlation Analyses

**Figure 3:**
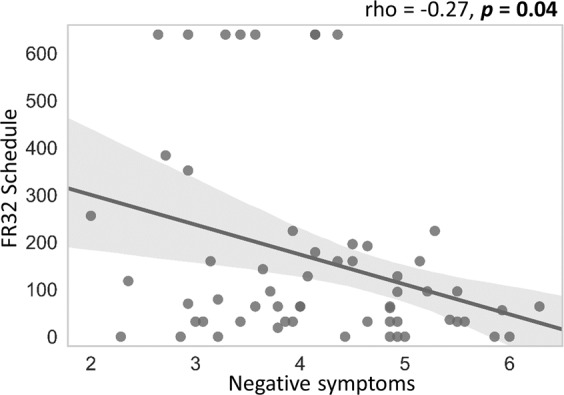
Correlation between negative symptoms and the FR32 schedule in the concurrent task.

Across all participants, *positive* symptoms positively correlated with the slope values of temporal discounting tasks for both food (r = 0.39, *p* < 0.01) and money (r = 0.30, *p = *0.02) meaning that higher positive symptoms were associated with lower discounting of delayed rewards across time. Furthermore, participants with higher *negative* symptoms exhibited less willingness to work to obtain the snack food in the concurrent task (correlation between negative symptoms and the number of key presses for the snack option in the FR32 schedule: rho = −0.27, *p* = 0.04). See Fig. 3. In addition, *depressive* symptoms and participants’ effort performance in the FR32 schedule resulted inversely correlated (rho = −0.31, *p* = 0.01). However, this negative correlation was not statistically significant after controlling for age (rho = −0.25, *p* = 0.06).

For detailed correlational analyses and analyses on hyperbolic parameter k, see Table S1 (Supplementary Materials).

## Discussion

The goals of this study were to examine: i) whether alterations in temporal and effort discounting are present in individuals with subclinical psychotic symptoms and, if so, ii) whether such alterations are associated with the severity of the negative symptoms. We found that, *first*, individuals with high levels of subclinical psychotic symptoms exhibit altered temporal discounting compared to individuals with medium and low levels of subclinical psychotic symptoms. *Secondly*, they liked food rewards similarly to the other groups and result willing to exert the required effort to obtain them. Only when the effort required was high, individuals with high PS were different from the other groups at the trend level. Interestingly, individuals who were less willing to exert effort in the higher effort condition were those with higher levels of negative symptoms, confirming a link between negative symptoms and effort discounting found in previous studies. Broadly speaking, these results are in line with studies showing alterations in temporal and effort computations in patients with schizophrenia^9,15,34^. On the other hand, some of our findings are at variance with these studies, as well as with our expectations. First, individuals with high levels of subclinical psychotic symptoms failed to exhibit a steeper discounting rate compared to controls. Secondly, discounting computations did not correlate with the severity of negative symptoms. We discuss results concerning temporal and effort discounting below.

Individuals with high levels of subclinical psychotic symptoms did not exhibit a steeper discounting rate compared to controls when we computed the AUC values. Interestingly, when we analyzed datasets using curve fitting, we found that, even if the hyperbolic model provides a good description of the data in accordance with the literature (e.g.^35^), the linear model showed overall a better fit. Interestingly, we observed that, while in individuals with medium and low PS both the linear model and the hyperbolic model fit the data well with both food and money, in individuals with high PS only the linear model provided an adequate description of the data with food rewards. When the slope discounting values were analyzed, we found that in participants with high PS the rate of discounting appeared linear, and flatter than that exhibited by participants with medium and low PS, especially with food rewards. This means that individuals with high PS were less sensitive to changes in discounting rates and temporal delays. Indeed, while medium PS and low PS individuals’ subjective values decreased as time delays increased, high PS exhibit lower subjective values of rewards when time delays were short (e.g. 2 days, 2 weeks) compared to the other two groups, and these values remain in general constant as time delays increased. In addition, slope values did not correlate with negative symptoms. However, this correlation was found when we considered hyperbolic parameter k (supplementary information), an aspect that deserves future investigation. These results are only partly in line with evidence that schizophrenia patients prefer more immediate rather than delayed rewards compared to healthy controls^10–13^. We should however mention that the results of the majority of the study on patients were based on the hyperbolic model and used monetary rewards.

Apart from these considerations, the findings concerning temporal discounting seem to suggest that even individuals with subclinical psychotic symptoms may experience alterations in the representation of value of rewards. In support of this idea, studies comparing brain structure and function in individuals with subclinical psychotic experiences and healthy controls found significant differences in grey matter volume in regions associated with reward, such as the anterior cingulate regions and insula^36^ as well as reduced activity in the ventral striatum^37^. However, delay discounting likely results from various psychological processes like time perception^38^, working memory^39^ and intelligence^40^ among others, which need to be considered in future investigations. Moreover, it is interesting to note that individuals with high PS tend to discount linearly food more than money (as the analysis on model fitting shows). As mentioned earlier, in the present study the decision to use food rewards was mainly based on the concurrent scheduled task. However, this result shows that assessing temporal and effort discounting of reward could be extremely informative, as it may be possible that in this population, individuals may be more willing to wait or exert effort for some but not for all rewards.

In examining effort computations, we found that individuals with high PS show similar liking scores for food rewards compared to individuals with medium PS and low PS, and were willing to exert the required effort to obtain them. Only when the effort required was high, high PS were different from the other groups at the trend level. The absence of a significant difference between groups across the whole task may be driven by the characteristics of the task we used, in which temporal confounds are not excluded (see^41^). Future studies should use a more valid version of the effort task, maybe extending observations also to cognitive effort. Physical and cognitive effort have been found to share a common network of areas, such as the dorsolateral prefrontal cortex among others^41,42^ and patients with schizophrenia have been found to be less likely to select a hard task (either physical or cognitive) associated with a large reward compared to an easy task associated with a smaller reward^43^.

Although this difference did not reach statistical significance, it may suggest that some alterations in effort may be present in individuals with subclinical psychotic experience. Specifically, this finding seems to suggest that anticipatory but not consummatory pleasure may be altered in individuals with subclinical psychotic symptoms. This is in agreement with results observed in schizophrenia patients showing that patients’ self-reported levels of pleasure are similar to those of healthy controls when experiencing pleasurable activities^44^; but, unlike healthy controls, they are less likely to select a hard task associated with a large reward compared to an easy task associated with a smaller reward^43^. Not only was our result marginally significant, but we did not observe difference between groups in anticipatory pleasure when we analyzed the results of the TEPS, a questionnaire developed to address anticipatory and consummatory pleasure in schizophrenia^45^. However differences between anticipatory and consummatory pleasure have not always been observed in schizophrenia patients^25,46,47^, this suggests that future studies should investigate effort computations in individuals with subclinical psychotic experience employing multiple valid measures of effort. Moreover, since different profiles or sub-types of psychosis can be identified, with patients often showing a prevalence of either positive or negative symptoms, it would be interesting in the future to assess temporal and effort cost decision making in both sub-types enrolling a larger sample of participants.

As a final remark, we found that alterations in reward processing were present only in individuals identified with the higher CAPE cut-off score. As pointed out in the Materials and Methods section, differences exist between the two cut-offs, in that they have different levels of sensitivity, specificity, and positive and negative predictive values^33^. Our study suggests that using the higher cut-off is preferable, especially since it is also the one with the higher specificity and low number of false positives^33^, although further research is needed.

Lastly, some limitations of the current study should be addressed. First, as we mentioned earlier, despite the usefulness of concurrent schedule task has been widely proven in animal research, this task has the disadvantage that effort costs may be confounded by temporal costs. In the present study, correlational analyses did not show any significant association with the temporal discounting task (see supplementary Table S2); nevertheless, future studies should carefully control for this aspect. Another limitation of the current study refers to the use of the CAPE cut-offs. Cut-off values are indeed calculated on the positive dimension and do not take into account negative scores^33^. However, we must acknowledge that the CAPE positive symptoms subscale has been shown to detect psychotic experiences and predicts psychotic illness better than the whole 42-item scale^48^.

In conclusion, our results are (at least in part) in line with studies conducted with schizophrenia patients, and extend current knowledge by showing that aberrant temporal and effort discounting of rewards are observed in healthy individuals with subclinical psychotic symptoms and therefore might be a vulnerability marker for psychosis. Identifying vulnerability markers for psychosis is of fundamental importance for early detection and treatment of psychosis.

## Materials and Methods

### Participants

An initial sample of 334 participants, mainly students at the University of Trieste, were contacted through a social network. They filled out an internet based questionnaire assessing subclinical psychotic symptoms (CAPE)^33^. The CAPE is a 42-item questionnaire that measures positive, negative and depressive symptoms and it has been proved to be a reliable and valid measure for self-reported subclinical psychotic symptoms in the general population^49^. From the above-mentioned sample, 60 participants were selected. The exclusion criteria were: (1) no personal history of neurological or psychiatric illness; (2) no family history of psychiatric illness in first-degree relatives; (3) no use of illicit substances (see^31^), (4) no eating disorders (assessed with the Eating disorder Inventory questionnaire, EDI-3)^50^; and no specific food restrictions (e.g., vegetarianism, allergy and others). All participants had a normal intelligence quotient measured using the Test di Intelligenza Breve (TIB)^51^ (mean: 106.2; SD: ±3.49; range: 95.72–112.74).

Since two CAPE cut-off scores have been proposed to identify individuals at-risk for psychosis (respectively, above 2.8 and 3.2 in the positive dimension sub-scale) and they have different levels of sensitivity, specificity, positive and negative predictive values^33^, in our study, we decided to use both of them and we divided participants into three groups. Specifically, 19 individuals with “high” PS, CAPE score > 3.2), 21 with “medium” PS (2.8 ≤ CAPE ≤ 3.2) and 20 with “low” PS (CAPE score < 2.8) were recruited. They were matched according to age, education and BMI. Written informed consent was obtained from all of them. All subjects were paid for their participation. The SISSA Ethics Committee approved the study, which was conducted in accordance with the Declaration of Helsinki.

### Procedure

Participants were instructed to refrain from eating at least 2 hours before attending the experiment, in order to induce similar levels of hunger across subjects. Participants’ subjective ratings of hunger were also collected, in order to control for macroscopic differences among subjects. We collected participants’ weight and height, and calculated body mass index (BMI) by dividing weight in kilograms by the square of height in meters (kg/m2). Participants were asked to perform, in a random order, two temporal discounting tasks and a concurrent schedule task. At the end of the experimental session, they underwent some neuropsychological tests and completed several questionnaires.

### Temporal discounting Tasks (temporal cost decision making)

Participants performed two computerized temporal discounting tasks in which they made choices between an amount of reward that could be received immediately (immediate option) and an amount of reward that could be received after some specific delays (delayed option)^52^. Specifically, after the presentation of a fixation cross (1 s), a choice between the immediate and the delayed option was presented. In each choice the amount of the reward, the image of the reward and the delay of delivery were displayed. Participants made their choices by pressing one of two buttons (see Fig. 1). After that, the chosen option remained on the screen for 1 s. The inter-trial interval was 1.5 s. Participants were asked to make five choices per block. Each block corresponded to a specific delay: 2 days, 2 weeks, 1 month, 3 months, 6 months and 1 year. An adjusting amount procedure was used across blocks to determine participants’ discounting rates. More specifically, the first choice presented to participants was always between 20 units for the immediate option and 40 units for the delayed one. During the task, the delayed option remained fixed; on the other hand, the immediate option was adjusted using a staircase procedure^53^. Thus, when the immediate option was chosen, in the following choice the amount of this option was decreased by half of the difference between the delayed and the immediate option. Conversely, when the delayed option was chosen, in the following choice the amount of the immediate option was increased. The size of the adjustment of the immediate option during trials was always half of the previous adjustment. After five choices for a specific delay, the participant began a new series of choices with another delay. The subjective value for each block was estimated as the immediate amount that would have been presented on the sixth trial. In one task, we used food (primary reward) while in another we used money (secondary reward). All rewards were hypothetical. For what concerns the food task, participants were asked to choose their preferred food from a selection of six popular snacks (to increase task ecological validity, snacks usually present in the vending machines were used as stimuli).

### Concurrent schedule task (effort cost decision making)

Before the concurrent schedule task, participants performed a liking-rating task in order to select food stimuli. Specifically, they were presented with eight real food items, four of which were high-calorie items (snack foods: Tuc, Kit-Kat, Kinder-Bueno and peanuts) and four were low-calorie items (fruits and vegetables: corn, carrots, strawberry yogurt and oranges) that had been previously selected through a questionnaire filled out by an independent sample (n = 118). Participants were asked to taste each food and subsequently rate their in-the-moment liking on a 11-point line Likert scale ranging from 0 (not at all) to 10 (extremely liked). Two favorite foods, one high-calorie and one low-calorie food, which did not differ more than one point on the 11-point Likert scale, were selected as stimuli in the experimental task (for one subject the difference was two points). For a similar procedure see^54^.

Following the liking-rating task, participants completed the concurrent schedule task. They were instructed that the task goal was to earn points to obtain two foods selected by the computer among those presented in the taste test. Specifically, they were asked to press a left or right key in correspondence with the part of the screen that displayed the food they wanted to earn a point for. However, they were also instructed that, as the task proceeded, it would become harder to get points for one of the two foods and that at the end of the experiment the total amount of food points obtained would be converted into the same amount of the respective food (in grams) and given to them. The task consisted of five schedules and each schedule comprises 20 choices. In the first schedule, the reinforcement ratio for both the snack option and the fruit or vegetable option was set at FR2: a point was earned every two presses on the same key for the corresponding food. The number of button presses for the snack option doubled every schedule (FR2, FR4, FR8, FR16, FR32). Thus, in the last schedule (FR32) participants were asked to press a key twice to obtain a fruit or vegetable point and 32 times on the other response key to earn one point for snacks (see Fig. 2). Allocation of the images (high-calories item vs. low-calories item) to the side of the screen (left vs. right) was counterbalanced across participants. One subject did not perform this task due to technical problems.

### Questionnaires and Neuropsychological tests

Participants were asked to complete the following questionnaires: the Behavioral Inhibition & Activation Scales (BIS/BAS)^55^, the Temporal Experience of Pleasure Scale (TEPS)^45^, the Barratt Impulsiveness Scale (BIS-11)^56^ and the Beck Depression Inventory^57^. Furthermore, participants performed the following neuropsychological tests assessing working memory and executive functions: Digit Span Forward test^58^ and Stroop Color Word Test (SCWT)^59^.

### Statistical Analysis

Data were analyzed using Statistica 8.0 (StatSoft, USA) software. The Kolmogorov-Smirnov test was undertaken to demonstrate that data were normally distributed. Comparisons between groups on demographic, questionnaire and neuropsychological measures were performed using one-way analyses of variance (ANOVA). Gender distribution was analyzed using χ2 test. For temporal discounting tasks, we assessed which of three different models (linear, hyperbolic and exponential) fit the data better by focusing on *R*^2^ scores^60^. Next, the rate at which the subjective value of a reward decays with delay was assessed through two indices: the area under the empirical discounting curve (AUC)^61^ and the slope of the regression line between subjective value and time intervals. In more detail, a negative slope value means that subjective values decrease as time delays increase, while a positive slope value means the opposite. A slope value equal to zero means that the participant’s subjective value is the same for each delay. Thus, higher scores on this index indicate lower discounting.

A repeated-measures analysis of variance (ANOVA) on AUC values was performed with Task (Food/Money) as a within-subjects variable and Group (high PS/medium PS/low PS) as a between-subjects variable. The same analysis was performed on the slope values. In the Liking task, a repeated-measures ANOVA on rating scores was performed with type of food (high-calorie/low-calorie) as a within-subjects variable and Group (high PS/medium PS/low PS) as a between-subjects variable. In the concurrent task, the rate at which participants are willing to work for snack foods was assessed through two indices: the number of key presses for the snack option for each schedule (FR2/FR4/FR8/FR16/FR32) and the individual slope, representing the trend of participants’ effort. Comparisons among groups in this task were performed using Mann–Whitney U-test.

## Supplementary information

Supplementary info

## Data Availability

The datasets generated during and/or analyzed during the current study are available from the corresponding authors on reasonable request.
