# Supplementary material for: Temporal and Effort cost Decision-making in Healthy Individuals with Subclinical Psychotic Symptoms
Source: Sci Rep. 2019 Feb 15;9:2151. doi: 10.1038/s41598-018-38284-x (PMC6377635; doi:10.1038/s41598-018-38284-x)
Supplement: Supplementary file 1 — Supplementary info [file 41598_2018_38284_MOESM1_ESM.pdf]

Temporal and Effort cost Decision-making in Healthy Individuals with Subclinical Psychotic Symptoms

Terenzi Damiano[1] , Mainetto Elena[1] , Barbato Mariapaola[2], Rumiati Raffaella[1,3] & Aiello Marilena[1,*]

Comparison between groups

Individuals with high PS, medium PS and low PS were matched for gender [$\chi 2$ (2) = 5.3, $p = 0.07$], age ($F_{2, 57} = 0.24$, $p = 0.78$), education ($F_{2, 57} = 0.33$, $p = 0.72$), and BMI ($F_{2, 57} = 1.23$, $p = 0.29$). In addition, they did not significantly differ on subjective ratings of hunger ($F_{2, 57} = 1.11$, $p = 0.34$).

CAPE scores

The one-way ANOVA on **frequency of positive symptoms** showed a main effect of group ($F_{2, 57} = 57.77$, $p < 0.0001$). Post-hoc analysis showed that both individuals with high PS and medium PS scored higher on this sub-scale compared to those with low PS. In addition, individuals with high PS scored higher compared to those with medium PS (all *ps* < 0.0001).

The ANOVA on **distress of positive symptoms** showed a main effect of group ($F_{2, 57} = 52.08$, $p < 0.0001$). Post-hoc analysis showed that both individuals with high PS and medium PS scored higher on this sub-scale compared to those with low PS. In addition, individuals with high PS scored higher compared to those with medium PS (all *ps* < 0.001).

The ANOVA on **total positive symptoms** yielded a main effect of group ($F_{2, 57} = 78.25$, $p < 0.0001$). Post-hoc analysis showed that both participants with high PS and medium PS scored higher on this sub-scale compared to those with low PS. In addition, participants with high PS scored higher compared to those with medium PS (all *ps* < 0.0001).

The ANOVA on **frequency of negative symptoms** showed a main effect of group ($F_{2, 57} = 2.57$, $p < 0.0001$). Post-hoc analysis showed that both individuals with high PS and medium PS scored higher on this

sub-scale compared to those with low PS (all *ps* < 0.01). Furthermore, participants with high PS scored higher compared to those with medium PS ($p = 0.04$).

The ANOVA on **distress of negative symptoms** showed a main effect of group ($F_{2, 57} = 13.24$, $p < 0.0001$). Post-hoc analysis showed that both individuals with high PS and medium PS scored higher on this sub-scale compared to those with low PS (all *ps* < 0.01). Furthermore, participants with high PS scored higher compared to those medium PS ($p = 0.05$).

The ANOVA on **total negative symptoms** scores showed a main effect of group ($F_{2, 57} = 13.53$, $p < 0.0001$). Post-hoc analysis showed that both individuals with high PS and medium PS scored higher on this sub-scale compared to those with low PS (all *ps* < 0.01). Moreover, high PS scored higher compared to medium PS ($p = 0.04$).

The ANOVA on **frequency of depressive symptoms** showed a main effect of group ($F_{2, 57} = 8.49$, $p < 0.001$). Post-hoc analysis showed that both individuals with high PS and medium PS scored higher on this sub-scale compared to those low PS ($p < 0.001$ and $p = 0.02$, respectively). However, no statistical differences emerged between individuals with high PS and medium PS ($p = 0.07$).

The ANOVA on **distress of depressive symptoms** showed a main effect of group ($F_{2, 57} = 12.35$, $p < 0.0001$). Post-hoc analysis showed that both individuals with high PS and medium PS scored higher on this sub-scale compared to those low PS (all *ps* < 0.001). However, no statistical differences emerged between individuals with high PS and medium PS ($p = 0.1$).

The ANOVA on **total depressive symptoms** showed a main effect of group ($F_{2, 57} = 12.89$, $p < 0.0001$). Post-hoc analysis showed that both individuals with high PS and medium PS scored higher on this sub-scale compared to those low PS (all *ps* < 0.001). However, no statistical differences emerged between individuals with high PS and medium PS ($p = 0.07$).

Other questionnaires and tests

There was no main effect of Group on BDI-II scores ($F_{2, 57} = 2.53$, $p = 0.09$) nor on BIS-11 total scores ($F_{2, 57} = 1.84$, $p = 0.17$) and on BIS-11 attentional ($F_{2, 57} = 1.89$, $p = 0.16$), motor ($F_{2, 57} = 2.37$, $p = 0.10$) and non-planning ($F_{2, 57} = 0.59$, $p = 0.56$) sub-scales. In addition, there was no main effect of Group on TEPS anticipatory ($F_{2, 57} = 0.44$, $p = 0.65$) and consummatory ($F_{2, 57} = 1.57$, $p = 0.22$) sub-scales. Furthermore, there

was no main effect of Group on Digit Span forward test ($F_{2, 57} = 0.28$, $p = 0.76$) and on Stroop Test for both performance time ($F_{2,57} = 0.32$, $p = 0.73$) and number of errors ($F_{2, 57} = 0.17$, $p = 0.84$).

Concurrent Schedule Task

Participants did not differ on number of key presses for the snack option in the following Schedules: **FR2** (high PS vs low PS: *U* = 166.5, Z = -0.41, *p* = 0.68; high PS vs medium PS: *U* = 188.5, Z = 0.3, *p* = 0.77; medium PS vs low PS: *U* = 182, Z = 0.5, *p* = 0.62), **FR4** (high PS vs low PS: *U* = 165, Z = 0.45, *p* = 0.65; high PS vs medium PS: *U* = 198.5, Z = 0.02, *p* = 0.98; medium PS vs low PS: *U* = 186, Z = -0.36, *p* = 0.71), **FR8** (high PS vs low PS: *U* = 166.5, Z = -0.41, *p* = 0.68; high PS vs medium PS: *U* = 175, Z = -0.66, *p* = 0.51; medium PS vs low PS: *U* = 194, Z = -0.15, *p* = 0.88) and **FR16** (high PS vs low PS: *U* = 157, Z = -0.69, *p* = 0.49; high PS vs medium PS: *U* = 149, Z = -1.37, *p* = 0.17; medium PS vs low PS: *U* = 179.5, Z = -0.54, *p* = 0.59). However, in the **FR32** schedule the number of key presses was lower for individuals with high PS group compared to those with low PS (*U* = 114, Z = -1.94, *p* = 0.052). There were no differences in this schedule between individuals with high PS and medium PS, only a marginal trend (*U* = 132.5, Z = -1.81, *p* = 0.07) and between individuals with medium PS and low PS (*U* = 196, Z = 0.09, *p* = 0.92). Furthermore, there were no differences on Slope values between the three groups (only a marginal difference for individuals with high PS vs medium PS: *U* = 130, Z = -1.88, *p* = 0.06; high PS vs low PS: *U* = 127, Z = -1.56, *p* = 0.12; medium PS vs low PS: *U* = 196, Z = 0.09, *p* = 0.92).

Temporal discounting task

The ANOVA on log-transformed *k* values with Group (high PS, medium PS, low PS) x Task (food, money) yielded a significant main effect of Task ($F_{1, 57} = 15.48$, $p < 0.001$), meaning a steeper temporal discounting for food than money. No other significant results emerged (*Ps* > 0.3).

Correlational analyses

Table S1. Correlation Analyses

	Positive symptoms	Negative symptoms	Depressive symptoms
**TD**			
AUC food	0.03	-0.09	-0.20
AUC money	0.14	0.02	0.05
slope food	**0.38****	0.16	0.14
slope money	**0.30***	0.12	0.11
log k food	0.17	**0.30***	0.20
log k money	-0.00	0.03	0.15
**Concurrent**			
FR32 schedule	-0.25	**-0.27***	**-0.31***
slope	-0.21	-0.23	-0.21

* = $p < 0.05$
** = $p < 0.01$

Table S2. Correlation Analyses between experimental tasks.

	FR32 schedule	***Slope (concurrent)***
AUC food	rho = 0.13; p = 0.31	rho = 0.12; p = 0.36
AUC money	rho = 0.10; p = 0.44	rho = 0.12; p = 0.47
Slope food	rho = -0.08; p = 0.56	rho = -0.06; p = 0.66
Slope money	rho = -0.06; p = 0.66	rho = -0.04; p = 0.77
